# Prognostic value of NT‐proBNP in patients with primary mitral regurgitation undergoing transcatheter edge‐to‐edge repair

**DOI:** 10.1002/ejhf.3725

**Published:** 2025-06-18

**Authors:** Philipp von Stein, Jessica Weimann, Roman Pfister, Sebastian Ludwig, Benedikt Koell, Erwan Donal, Dhairya Patel, Lukas Stolz, Tetsu Tanaka, Andrea Scotti, Teresa Trenkwalder, Felix Rudolph, Daryoush Samim, Cristina Giannini, Julien Dreyfus, Jean‐Michel Paradis, Marianna Adamo, Nicole Karam, Yohann Bohbot, Anne Bernard, Bruno Melica, Angelo Quagliana, Yoan Lavie Badie, Mirjam Kessler, Omar Chehab, Simon Redwood, Edith Lubos, Lars Sondergaard, Marco Metra, Chiara Primerano, Fabien Praz, Muhammed Gerçek, Erion Xhepa, Georg Nickenig, Azeem Latib, Niklas Schofer, Raj Makkar, Juan F. Granada, Thomas Modine, Jörg Hausleiter, Augustin Coisne, Daniel Kalbacher, Christos Iliadis, on behalf of the PRIME‐MR Investigators, Paul Achouh, David Attias, Stephan Baldus, Alain Berrebi, Guillaume Bonnet, Laura Granada, Frederic Bouisset, Corentin Bourg, Thierry Bourguignon, Diogo Ferreira, Stéphane Lafitte, Thibault Lhermusier, Guillaume L'official, Mohammed Nejjari, Edoardo Pancaldi, Lydia X. Plewe, Tobias Rheude, Natacha Rousse, Volker Rudolph, Dan Rusinaru, Christophe Saint‐Etienne, Francisco Sampaio, Jury Schewel, Arnaud Sudre, Christophe Tribouilloy, Marcel Weber, Stephan Windecker

**Affiliations:** ^1^ Department III of Internal Medicine, Faculty of Medicine and University Hospital Cologne University of Cologne Cologne Germany; ^2^ Cardiovascular Research Foundation New York NY USA; ^3^ Department of Cardiology, University Heart & Vascular Center Hamburg University Medical Center Hamburg‐Eppendorf Hamburg Germany; ^4^ DZHK, German Center for Cardiovascular Research Partner Site Hamburg/Kiel/Lübeck Hamburg Germany; ^5^ Cardiology Department, CHU de Rennes Rennes France; ^6^ Smidt Heart Institute Cedars‐Sinai Medical Center Los Angeles CA USA; ^7^ Medizinische Klinik und Poliklinik I, Klinikum der Universität München Munich Germany; ^8^ German Center for Cardiovascular Research (DZHK), Partner Site Munich Heart Alliance Munich Germany; ^9^ Department of Internal Medicine II, Heart Center Bonn Bonn Germany; ^10^ Montefiore‐Einstein Center for Heart and Vascular Care, Montefiore Medical Center Albert Einstein College of Medicine New York NY USA; ^11^ Department of Cardiology, German Heart Center Munich Technical University of Munich Munich Germany; ^12^ Clinic for General and Interventional Cardiology/Angiology, Heart and Diabetes Center NRW Bad Oeynhausen Germany; ^13^ Universitätsklinik für Kardiologie, Inselspital Bern Bern Switzerland; ^14^ S.D. Emodinamica, AOUP ‐ Azienda Ospedaliero Universitaria Pisana Pisa Italy; ^15^ Cardiology Department Centre Cardiologique du Nord Paris France; ^16^ Quebec Heart & Lung Institute Laval University Quebec City QC Canada; ^17^ Cardiac Catheterization Laboratory and Cardiology, ASST Spedali Civili, and Department of Medical and Surgical Specialties, Radiological Sciences, and Public Health University of Brescia Brescia Italy; ^18^ Cardiology Department, European Hospital Georges Pompidou Paris France; ^19^ Department of Cardiology Amiens University Hospital Amiens France; ^20^ Cardiology Department, CHRU de Tours Tours France; ^21^ Centro Hospitalar Vila Nova de Gaia Espinho Portugal; ^22^ Rigshospitalet Copenhagen University Hospital Copenhagen Copenhagen Denmark; ^23^ Department of Cardiology Rangueil University Hospital Toulouse France; ^24^ Department of Internal Medicine II Ulm University Heart Center Ulm Germany; ^25^ Department of Cardiology, St. Thomas' Hospital London UK; ^26^ Marienkrankenhaus Hamburg Germany; ^27^ Service Médico‐Chirurgical: Valvulopathies‐Chirurgie Cardiaque‐Cardiologie Interventionelle Structurelle Centre Hospitalier Universitaire Bordeaux Bordaux France; ^28^ Department of Clinical Physiology and Echocardiography ‐ Heart Valve Clinic, CHU Lille Lille France

**Keywords:** Primary mitral regurgitation, Mitral valve transcatheter edge‐to‐edge repair, NT‐proBNP, PRIME‐MR

## Abstract

**Aims:**

The prognostic value of N‐terminal pro‐B‐type natriuretic peptide (NT‐proBNP) in patients undergoing mitral valve transcatheter edge‐to‐edge repair (M‐TEER) for primary mitral regurgitation (PMR) is unclear. This study assessed the association between NT‐proBNP and outcomes and explored its additive value to the Mitral Regurgitation International Database (MIDA) score.

**Methods and results:**

PRIME‐MR, a retrospective, international, multicentre registry, includes 3083 consecutive PMR patients treated with M‐TEER. This analysis focused on 1382 patients (median age 81 years, 47% female, 82% New York Heart Association [NYHA] functional class III/IV, median EuroSCORE II 4.1%) with available NT‐proBNP levels and follow‐up. The primary endpoint was death or heart failure hospitalization within 3 years. Median NT‐proBNP level was 1991 pg/ml (T1: 578, T3: 6285), and 384 patients reached the primary endpoint (Kaplan–Meier estimate: 48.5%). Log‐transformed NT‐proBNP levels independently predicted the primary endpoint (adjusted hazard ratio [HR] 1.17, 95% confidence interval [CI] 1.07–1.28; *p* < 0.001) after adjusting for NYHA class, haemoglobin, creatinine, and atrial fibrillation. In 1041 patients with a modified MIDA score (median 9), the score was initially associated with the primary endpoint (HR 1.10, 95% CI 1.04–1.17; *p* = 0.002), but lost significance when adjusting for NT‐proBNP levels, which remained independently predictive (adjusted HR 1.20, 95% CI 1.07–1.34; *p* = 0.002).

**Conclusions:**

NT‐proBNP, but not the MIDA score, was independently associated with death or heart failure hospitalizations within 3 years in M‐TEER‐treated PMR patients. Incorporating NT‐proBNP levels into clinical assessment may improve risk stratification and potentially supports earlier intervention at lower NT‐proBNP levels to optimize outcomes.

## Introduction

Primary mitral regurgitation (PMR), a disease of the mitral valve (MV) apparatus, accounts for approximately one‐third of patients with clinically significant mitral regurgitation (MR), the most prevalent valvular heart disease worldwide.^1^ Symptomatic PMR is associated with a poor prognosis, characterized by excess mortality and heart failure (HF). While surgical MV repair is the treatment of choice, many PMR patients are ineligible due to high surgical risk.^2^, ^3^ To address this unmet treatment need, MV transcatheter edge‐to‐edge repair (M‐TEER) has been established as an effective and safe alternative for high‐risk patients.^4^ However, variability in symptom relief and long‐term outcomes remains a major challenge in this highly morbid and elderly patient population, for whom established prognostic markers are lacking.

B‐type natriuretic peptides, such as N‐terminal pro‐B‐type natriuretic peptide (NT‐proBNP), are released in response to myocardial wall stress and are well‐established biomarkers for the diagnosis and risk stratification of HF, correlating with disease severity and adverse cardiovascular outcomes.^5^ Reflecting cumulative cardiac compromise—including valvular‐induced stress, cardiac damage due to delayed treatment, and valvular‐independent cardiac conditions—NT‐proBNP may serve as a valuable prognostic marker in PMR patients undergoing M‐TEER. However, its predictive value in this context is unclear.

The Mitral Regurgitation International Database (MIDA) surgical risk score is well‐established for risk stratification in PMR patients managed conservatively or undergoing surgical MV repair.^6^ Yet, its applicability to those undergoing M‐TEER has been explored only in a small M‐TEER subpopulation.^7^ However, its inclusion of several pathophysiologically relevant parameters for PMR, makes it a potentially valuable tool in this context. Notably, the MIDA score does not incorporate biomarkers; therefore, integrating NT‐proBNP could potentially refine its predictive accuracy and enhance risk stratification.

Given the lack of established parameters for outcome prediction in PMR patients undergoing M‐TEER, this study aimed to investigate the association of pre‐interventional NT‐proBNP levels and the MIDA score with clinical outcomes using data from the global multicentre PRIME‐MR registry (Outcomes of Patients tReated wIth Mitral Transcatheter Edge‐to‐edge Repair for Primary Mitral Regurgitation; NCT05332782).

## Methods

### Study population and data collection

The design and methodology of the PRIME‐MR registry have been previously detailed.^8^ In brief, PRIME‐MR is an international, non‐interventional, retrospective, investigator‐initiated multicentre registry that included consecutive PMR patients who underwent M‐TEER from 2008 to 2022. Patients were assessed as high or prohibitive surgical risk by local interdisciplinary heart teams and deemed eligible for M‐TEER based on clinical and anatomical criteria.

Data collection and statistical analyses were coordinated centrally at the Centre for Population Health Innovation, University Heart and Vascular Centre Hamburg, University Medical Centre Hamburg‐Eppendorf, Hamburg, Germany. Follow‐up was conducted by local investigators through clinical visits, phone interviews, and record reviews. The study complied with the Declaration of Helsinki, received institutional review boards approval, and obtained written informed consent from all patients. Patients with missing baseline NT‐proBNP levels or survival data were excluded from this analysis.

### Echocardiographic assessment

Echocardiographic assessments were performed as previously described.^8^ In summary, site‐reported comprehensive assessments of transthoracic and transoesophageal echocardiograms included MR aetiology, severity, MV morphology, left atrial (LA) and left ventricular (LV) parameters, tricuspid annular plane systolic excursion (TAPSE), and echocardiographically estimated pulmonary artery systolic pressure (PASP).

### MIDA score assessment

The MIDA score comprises age ≥65 years (3 points), New York Heart Association (NYHA) functional class ≥II (3 points), atrial fibrillation (1 point), LA diameter ≥55 mm (1 point), PASP ≥50 mmHg (2 points), LV end‐systolic diameter ≥40 mm (1 point), and LV ejection fraction <60% (1 point).^6^ For this analysis, we adapted the score per the more recently published MIDA‐Q score,^9^ adjusting the NYHA class threshold to ≥III and replacing LA diameter ≥55 mm with an LA volume index of ≥60 ml/m^2^. The modified MIDA (mMIDA) score was stratified into tertiles.

### Endpoints

The primary endpoint was a composite of all‐cause mortality or HF hospitalization within 3 years, analysed as a time‐to‐first event. Secondary endpoints included the individual components of the primary endpoint, and functional outcomes by NYHA class were assessed at 1 year. Procedural and post‐procedural outcomes were evaluated according to the Mitral Valve Academic Research Consortium criteria.^10^

### Statistical analysis

Categorical variables were expressed as counts and proportions, compared using the *χ* ^2^ test. Continuous variables were presented as mean ± standard deviation or median (Q1–Q3), depending on the Shapiro–Wilk test results. Non‐normally distributed data were analysed using the Kruskal–Wallis test.

Patients were stratified into three groups based on baseline NT‐proBNP tertiles: T1 (1st tertile), T2 (2nd tertile), and T3 (3rd tertile). Median follow‐up time was estimated using the reverse Kaplan–Meier method. Kaplan–Meier estimates assessed freedom from the primary endpoint, with comparisons by the log‐rank test. The Benjamini–Hochberg correction was applied for multiple testing adjustments.

Cox proportional hazards regression models estimated the primary endpoint risk, with NT‐proBNP logarithmically transformed. Multivariable Cox regression analyses included parameters with a univariable significance level of <0.25, excluding those with >20% missing values or correlations >0.7. Stepwise backward selection based on Akaike's information criterion identified the optimal multivariable model, with NT‐proBNP forced into the model.

Sensitivity analyses included adjusted models for renal dysfunction, obesity (body mass index [BMI] >30 kg/m^2^), and isolated PMR.^11^ Additionally, we performed an exploratory sensitivity analysis using a dichotomized NT‐proBNP threshold of 1000 ng/L. The mMIDA scores were calculated as described, and multiple imputation (using 50 datasets with predictive mean matching) included patients with ≤2 missing parameters. Centres systematically lacking specific mMIDA data were excluded from imputation. A two‐sided *p* < 0.05 was considered statistically significant. All statistical analyses were performed using R, version 4.0.3 (The R Foundation for Statistical Computing, Vienna, Austria).

## Results

### Baseline characteristics

Out of 3083 patients in the PRIME‐MR registry, 1382 patients (median age: 81 years [76–85 years], 47% female, 82% in NYHA class III/IV, EuroSCORE II 4.1% [2.4–6.9%]) were included in this analysis. Baseline clinical and echocardiographic characteristics of included compared to excluded patients are presented in online supplementary *Tables Appendix* *S1* and *S2*.

**Figure 1:**
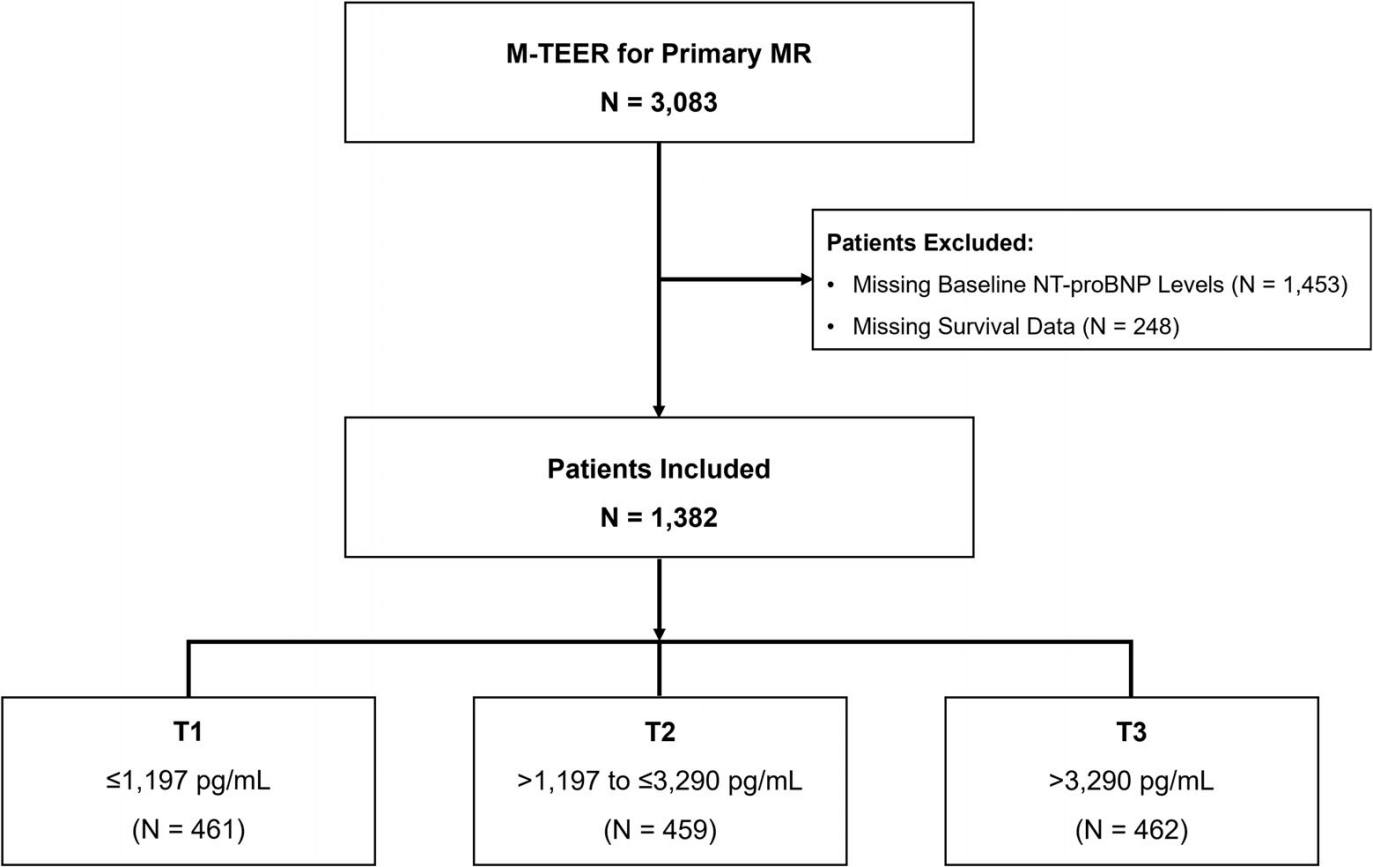
Study flowchart. The PRIME‐MR registry includes a total of 3083 patients. N‐terminal pro‐B‐type natriuretic peptide (NT‐proBNP) and survival status was available in 1382 patients. Patients were categorized into three groups according to NT‐proBNP tertiles. MR, mitral regurgitation; M‐TEER, mitral valve transcatheter edge‐to‐edge repair.

According to the study definition, patients were categorized by NT‐proBNP tertiles, as presented in *Figure* *1*. The median NT‐proBNP level was 1991 pg/ml (829–4260 pg/ml). Patients in higher NT‐proBNP tertiles were older, more symptomatic, and had higher surgical risk scores. They also demonstrated lower haemoglobin levels, reduced estimated glomerular filtration rates, and more frequent history of HF hospitalizations compared to lower tertiles (*Table* *1*). Baseline medications are detailed in online supplementary *Table* *S3*.

**Table 1:** Clinical baseline characteristics according to NT‐proBNP tertiles

	All (*n* = 1382)	1^st^ Tertile (*n* = 461)	2^nd^ Tertile (*n* = 459)	3^rd^ Tertile (*n* = 462)	*p*‐value
Demographic					
Age, years	81.0 (76.0–85.0)	80.0 (75.0–85.0)	81.0 (76.0–84.0)	82.0 (77.2–85.0)	**0.001**
Male sex	727 (52.6)	249 (54.1)	236 (51.4)	242 (52.4)	0.710
BMI, kg/m^2^	24.7 (22.1–27.7)	24.7 (22.2–28.0)	24.8 (22.0–27.9)	24.6 (22.1–27.3)	0.350
Surgical risk scores					
EuroSCORE II, %	4.1 (2.4–6.9)	2.9 (1.8–4.8)	4.1 (2.7–6.1)	5.5 (3.5–8.8)	**<0.001**
STS‐PROM (MV repair), %	3.8 (2.4–6.2)	3.2 (2.1–4.9)	3.7 (2.3–6.0)	5.0 (2.9–7.6)	**<0.001**
Comorbidities					
Arterial hypertension	1047 (76.4)	337 (74.1)	348 (76.1)	362 (79.0)	0.210
Diabetes mellitus	220 (16.1)	61 (13.4)	85 (18.6)	74 (16.1)	0.099
Coronary artery disease	593 (43.4)	169 (37.1)	191 (42.2)	233 (50.9)	**<0.001**
Atrial fibrillation	926 (67.1)	232 (50.4)	340 (74.1)	354 (76.6)	**<0.001**
Chronic lung disease	229 (16.6)	59 (12.8)	82 (17.9)	88 (19.0)	**0.026**
History of stroke	153 (11.1)	36 (7.9)	61 (13.3)	56 (12.2)	**0.022**
History of myocardial infarction	201 (15.0)	64 (14.3)	55 (12.4)	82 (18.4)	**0.036**
History of cardiac surgery	231 (16.7)	67 (14.5)	74 (16.1)	90 (19.5)	0.120
Clinical presentation					
NYHA class					
I	12 (0.9)	6 (1.3)	5 (1.1)	1 (0.2)	0.160
II	231 (17.0)	117 (26.3)	72 (15.9)	42 (9.1)	**<0.001**
III	904 (66.5)	272 (61.1)	320 (70.5)	312 (67.8)	**0.009**
IV	212 (15.6)	50 (11.2)	57 (12.6)	105 (22.8)	**<0.001**
Six‐min walking distance, m	215 (120.0–315.5)	289 (175.0–363.0)	242.5 (149.2–320.0)	159 (64.5–244.8)	**<0.001**
MLHFQ, points	34.0 (22.0–46.0)	29.5 (18.0–42.0)	31.5 (19.0–44.0)	37.0 (27.0–48.0)	**<0.001**
History of hospitalization for heart failure 12 months prior	388 (43.3)	103 (31.1)	139 (45.3)	146 (56.6)	**<0.001**
Laboratory results					
Haemoglobin, g/dl	12.3 (10.9–13.5)	12.7 (11.5–13.9)	12.4 (11.1–13.7)	11.8 (10.4–12.9)	**<0.001**
NT‐proBNP, pg/ml	1991 (828.8–4260)	578 (315–827)	1990 (1566–2607)	6285 (4260–10 053.8)	**<0.001**
Renal function					
Creatinine, mg/dl	1.2 (0.9–1.5)	1.0 (0.8–1.3)	1.1 (0.9–1.4)	1.4 (1.1–1.8)	**<0.001**
eGFR, ml/min	49.1 (36.0–65.6)	56.8 (43.9–72.7)	50.8 (38.6–66.0)	39.9 (30.6–54.8)	**<0.001**
On dialysis	20 (1.5)	1 (0.2)	2 (0.4)	17 (3.7)	**<0.001**

Values are *n* (%), median (Q1–Q3), or mean ± standard deviation.

BMI, body mass index; eGFR, estimated glomerular filtration rate; EuroSCORE, European System for Cardiac Operative Risk Evaluation; MLHFQ, Minnesota Living with Heart Failure Questionnaire; MV, mitral valve; NT‐proBNP, N‐terminal pro‐B‐type natriuretic peptide; NYHA, New York Heart Association; STS‐PROM, Society of Thoracic Surgeons predicted risk of mortality.

Echocardiographic data (*Table* *2*) showed that higher NT‐proBNP levels were associated with larger LA volumes, lower LV ejection fractions, and a lower prevalence of isolated PMR. Additionally, severe tricuspid regurgitation and higher PASP values, along with lower TAPSE and TAPSE/PASP ratios, were observed with increasing NT‐proBNP levels. MR severity was consistent across tertiles. MV prolapse was least frequent in T3, while mitral annular calcification including the MV leaflets, was most frequent in T3.

**Table 2:** Echocardiographic baseline characteristics according to NT‐proBNP tertiles

	All (*n* = 1382)	1^st^ Tertile (*n* = 461)	2^nd^ Tertile (*n* = 459)	3^rd^ Tertile (*n* = 462)	*p*‐value
Left atrium					
Left atrial volume, ml	111.0 (80.0–146.0)	100.0 (76.0–130.0)	117.0 (84.5–156.0)	120.0 (89.0–153.0)	**<0.001**
Left atrial volume index, ml/m^2^	66.0 (48.0–85.0)	59.0 (43.0–78.0)	68.5 (50.0–88.2)	70.0 (51.0–87.8)	**<0.001**
Left ventricle					
LVEF, %	58.0 (50.0–64.0)	60.0 (55.0–65.0)	57.0 (50.0–64.0)	55.0 (47.0–60.8)	**<0.001**
LVEDD, mm	53.0 (47.0–59.0)	52.0 (48.0–58.2)	53.0 (46.0–59.0)	52.5 (47.0–59.0)	0.970
LVESD, mm	36.0 (30.0–43.0)	35.0 (30.0–41.0)	35.5 (30.0–43.0)	38.0 (31.0–44.0)	**0.015**
LVEDV, ml	112.0 (86.0–145.0)	112.0 (89.3–146.0)	113.0 (88.0–141.0)	111.0 (82.2–146.8)	0.720
LVESV, ml	46.0 (33.9–66.0)	44.0 (32.0–62.0)	46.0 (35.0–62.7)	47.2 (33.8–73.5)	0.063
Mitral valve					
Mean transmitral gradient, mmHg	2.6 ± 1.3	2.5 ± 1.2	2.6 ± 1.3	2.8 ± 1.5	**0.005**
3D MVOA, cm^2^	4.4 (3.6–5.6)	4.4 (3.8–5.5)	4.7 (3.6–5.7)	4.3 (3.5–5.7)	0.510
AML length, mm	23.0 (20.0–27.0)	23.0 (20.0–27.0)	24.0 (20.1–28.0)	23.0 (20.0–26.8)	0.190
PML length, mm	13.0 (11.0–16.0)	14.0 (11.1–17.0)	13.0 (11.0–16.0)	13.0 (11.0–15.1)	**0.023**
Mitral annular calcification in 3D	437 (75.0)	163 (78.4)	143 (77.7)	131 (68.6)	**0.046**
Anterior ring	13 (2.2)	3 (1.4)	5 (2.7)	5 (2.6)	0.630
Posterior ring	53 (9.1)	21 (10.1)	12 (6.5)	20 (10.5)	0.340
Total annulus	22 (3.8)	5 (2.4)	9 (4.9)	8 (4.2)	0.410
Annulus including leaflets	31 (5.3)	5 (2.4)	6 (3.3)	20 (10.5)	**<0.001**
Mitral regurgitation					
EROA, cm^2^	0.4 (0.3–0.6)	0.4 (0.3–0.6)	0.4 (0.3–0.6)	0.4 (0.3–0.7)	0.900
Regurgitant volume, ml	64.0 (50.0–82.6)	65.0 (52.0–81.0)	64.0 (47.0–80.0)	66.0 (51.5–85.2)	0.790
Grade 3+	315 (24.0)	94 (21.7)	119 (27.4)	102 (22.9)	0.120
Grade 4+	999 (76.0)	340 (78.3)	316 (72.6)	343 (77.1)	0.120
Aetiology					
Isolated PMR	828 (81.7)	324 (89.3)	277 (79.8)	227 (74.9)	**<0.001**
Mixed, leading cause PMR	185 (18.3)	39 (10.7)	70 (20.2)	76 (25.1)	**<0.001**
Main pathology					
Flail	526 (47.4)	195 (49.4)	149 (40.7)	182 (52.1)	**0.006**
Flail gap, mm	5.0 (3.0–7.0)	5.0 (3.5–7.0)	4.1 (3.0–6.0)	5.0 (3.0–6.8)	0.051
Flail width, mm	10.0 (7.0–14.0)	11.0 (8.0–14.0)	9.0 (7.0–13.0)	10.0 (7.0–13.4)	**0.042**
Prolapse	503 (45.3)	185 (46.8)	184 (50.3)	134 (38.4)	**0.005**
Calcification	53 (4.8)	11 (2.8)	22 (6.0)	20 (5.7)	0.068
Segment of main pathology					
A1	33 (3.6)	8 (2.6)	10 (3.2)	15 (5.0)	0.240
A2	269 (29.3)	70 (22.5)	92 (29.8)	107 (35.9)	**0.001**
A3	37 (4.0)	10 (3.2)	12 (3.9)	15 (5.0)	0.520
P1	67 (7.3)	22 (7.1)	25 (8.1)	20 (6.7)	0.790
P2	435 (47.4)	169 (54.3)	150 (48.5)	116 (38.9)	**<0.001**
P3	69 (7.5)	31 (10.0)	18 (5.8)	20 (6.7)	0.120
Right ventricle					
Severe tricuspid regurgitation	277 (22.1)	59 (13.9)	91 (21.9)	127 (30.4)	**<0.001**
TAPSE, mm	19.0 (16.0–23.0)	21.0 (18.0–25.0)	19.0 (16.0–22.0)	17.0 (14.0–21.0)	**<0.001**
PASP, mmHg	48.0 (37.0–60.0)	42.3 (33.0–51.9)	49.0 (37.0–60.1)	52.9 (42.0–67.0)	**<0.001**
TAPSE/PASP ratio, mm/mmHg	0.4 (0.3–0.6)	0.5 (0.4–0.7)	0.4 (0.3–0.5)	0.3 (0.2–0.4)	**<0.001**

Values are *n* (%), median (Q1–Q3), or mean ± standard deviation.

AML, anterior mitral leaflet; EROA, effective regurgitant orifice area; LVEDD, left ventricular end‐diastolic diameter; LVEDV, left ventricular end‐diastolic volume; LVEF, left ventricular ejection fraction; LVESD, left ventricular end‐systolic diameter; LVESV, left ventricular end‐systolic volume; MVOA, mitral valve orifice area; NT‐proBNP, N‐terminal pro‐B‐type natriuretic peptide; PASP, pulmonary artery systolic pressure; PML, posterior mitral leaflet; PMR, primary mitral regurgitation; TAPSE, tricuspid annular plane systolic excursion.

### Procedural outcomes

Procedural outcomes were largely similar across NT‐proBNP tertiles (*Table* *3*). Device selection and iterations used are reported in online supplementary *Table* *S4*. MR reduction to ≤1+ at discharge was achieved in 68.4% of patients in T1, 68.5% in T2, and 51.7% in T3 (*p* < 0.001 across groups).

**Table 3:** Procedural outcomes according to NT‐proBNP tertiles

	All (*n* = 1382)	1^st^ Tertile (*n* = 461)	2^nd^ Tertile (*n* = 459)	3^rd^ Tertile (*n* = 462)	*p*‐value
Procedure					
Duration, min	86.0 (59.0–116.5)	86.0 (59.0–117.5)	85.0 (57.0–111.0)	86.0 (61.0–120.8)	0.660
Number of devices	1.0 (1.0–2.0)	1.0 (1.0–2.0)	1.0 (1.0–2.0)	1.0 (1.0–2.0)	0.310
Technical success	1271 (92.9)	426 (93.8)	423 (92.8)	422 (92.1)	0.600
Conversion to open heart surgery	13 (0.9)	4 (0.9)	6 (1.2)	3 (0.7)	0.570
Device embolization	3 (0.2)	2 (0.5)	1 (0.2)	0 (0)	0.370
SLDA	17 (1.4)	4 (1.0)	3 (0.7)	10 (2.5)	0.077
AML	14 (1.1)	3 (0.7)	3 (0.7)	8 (2.0)	0.160
PML	3 (0.2)	1 (0.2)	0 (0)	2 (0.5)	0.370
Post‐procedural outcomes					
Mortality	9 (0.7)	3 (0.7)	3 (0.7)	3 (0.7)	>0.999
Stroke	11 (0.9)	3 (0.8)	4 (1.0)	4 (1.0)	0.930
Cardiogenic shock	24 (2.3)	7 (1.9)	4 (1.1)	13 (4.0)	**0.037**
Major, extensive, or life‐threating bleeding	24 (2.1)	8 (2.0)	10 (2.6)	6 (1.7)	0.660
Major vascular complication	37 (2.9)	10 (2.3)	13 (3.1)	14 (3.3)	0.170
Acute kidney injury	42 (3.4)	7 (1.7)	15 (3.2)	20 (4.3)	0.056
Echocardiography at discharge					
Residual MR					
None/trace	160 (12.6)	74 (17.1)	57 (13.5)	29 (7.0)	**<0.001**
1+	630 (49.5)	223 (51.4)	221 (52.4)	186 (44.7)	0.054
2+	366 (28.8)	106 (24.4)	112 (26.5)	148 (35.6)	**<0.001**
3+	62 (4.9)	14 (3.2)	17 (4.0)	31 (7.5)	**0.010**
4+	54 (4.2)	17 (3.9)	15 (3.6)	22 (5.3)	0.420
Mean transmitral gradient, mmHg	4.0 ± 1.7	4.1 ± 1.8	3.9 ± 1.7	3.9 ± 1.7	0.510
≥5 mmHg	352 (30.9)	130 (33.2)	105 (28.0)	117 (31.5)	0.280

Values are *n* (%), median (Q1–Q3), or mean ± standard deviation.

AML, anterior mitral leaflet; MR, mitral regurgitation; NT‐proBNP, N‐terminal pro‐B‐type natriuretic peptide; PML, posterior mitral leaflet; SLDA, single leaflet device attachment.

### Endpoints

**Figure 2:**
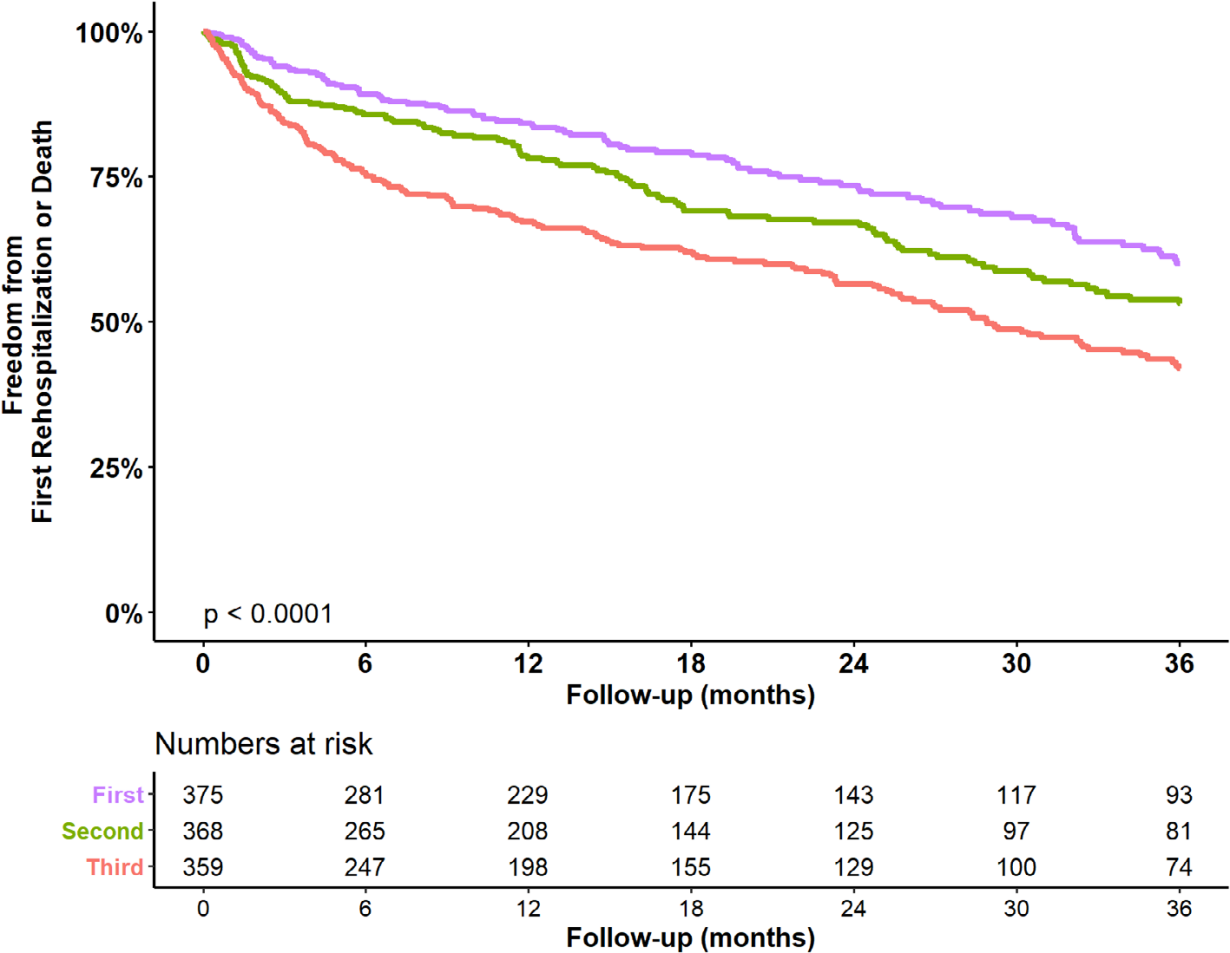
Kaplan–Meier survival curves for all‐cause mortality or heart failure hospitalization within 3 years stratified by N‐terminal pro‐B‐type natriuretic peptide (NT‐proBNP) tertiles (T1–T3). Significant differences were observed between T1 versus T2 (*p* = 0.042), T1 versus T3 (*p* < 0.001), and T2 versus T3 (*p* = 0.003).

Data on death and HF hospitalization were available for 1102 patients, over a median follow‐up of 2.41 years (95% confidence interval [CI] 2.13–2.76 years). Over the 3‐year period, 384 patients (48.5%) reached the primary endpoint, with higher rates in higher NT‐proBNP tertiles: 40.0% in T1, 46.8% in T2, and 58.1% in T3 (*p* < 0.001 across groups). Patients in the lowest tertile (T1) exhibited significantly lower event rates compared to those in T2 and T3; similarly, patients in T2 had significantly lower event rates than those in T3 (*Figure* *2*).

**Figure 3:**
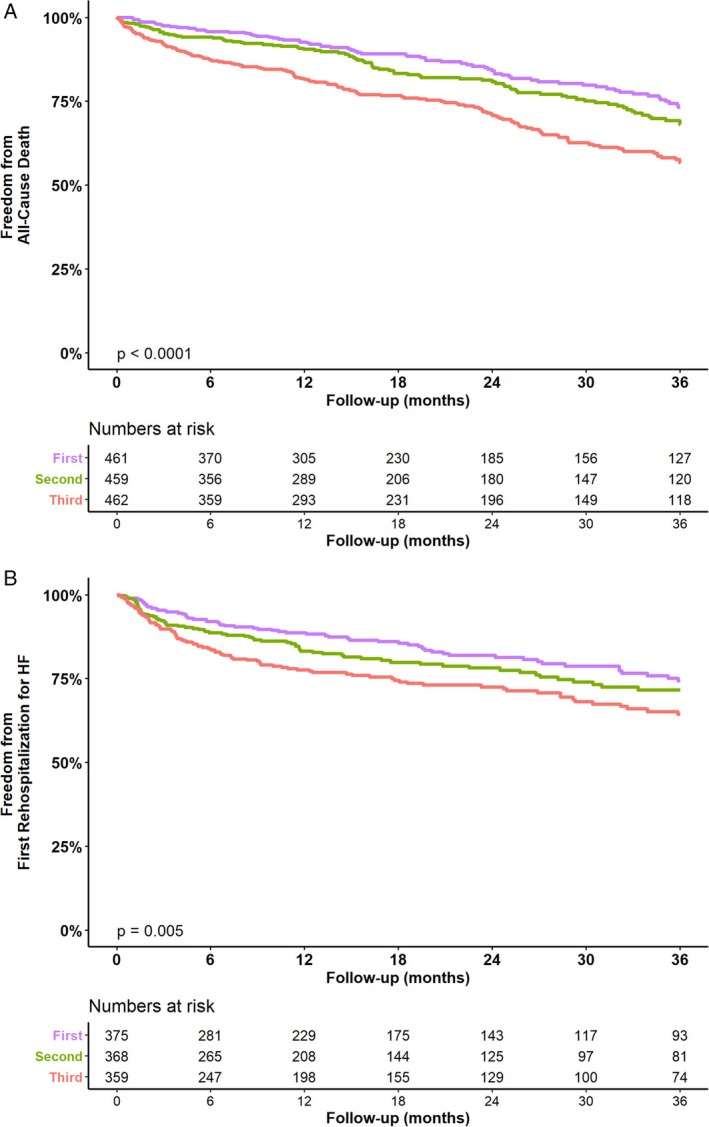
Kaplan–Meier survival curves for all‐cause mortality (*A*) and heart failure (HF) hospitalization (*B*) within 3 years stratified by N‐terminal pro‐B‐type natriuretic peptide (NT‐proBNP) tertiles (T1–T3). For all‐cause mortality, significant differences were observed between T1 versus T2 (*p* < 0.001) and T2 versus T3 (*p* < 0.001), but not for T1 versus T2 (*p* = 0.120). For HF hospitalizations, a significant difference was observed between T1 versus T3 (*p* = 0.0046). No significant differences were found between other tertile comparisons: T1 versus T2 (*p* = 0.19) and T2 versus T3 (*p* = 0.091).

At 3 years, 305 patients (34.4%) had died. All‐cause mortality rates increased with higher NT‐proBNP tertiles: 26.8% in T1, 31.9% in T2, and 43.3% in T3 (*p* < 0.001 across tertiles) (*Figure* *3A*). Post‐hoc analyses showed a significantly lower mortality in T1 and T2 versus T3 but not in T1 versus T2. HF hospitalizations at 3 years occurred in 226 patients (29.9%): 25.7% in T1, 28.4% in T2, and 35.7% in T3 (*p* = 0.005) (*Figure* *3B*). A significant difference was observed between T1 versus T3.

Functional outcomes, assessed by NYHA class at 1 year, showed that 355 patients (67.7%) achieved NYHA class ≤II. Rates varied significantly across NT‐proBNP tertiles: 73.9% in T1, 64.5% in T2, and 63.2% in T3 (*p* = 0.055).

### Cox regression and spline analysis

**Figure 4:**
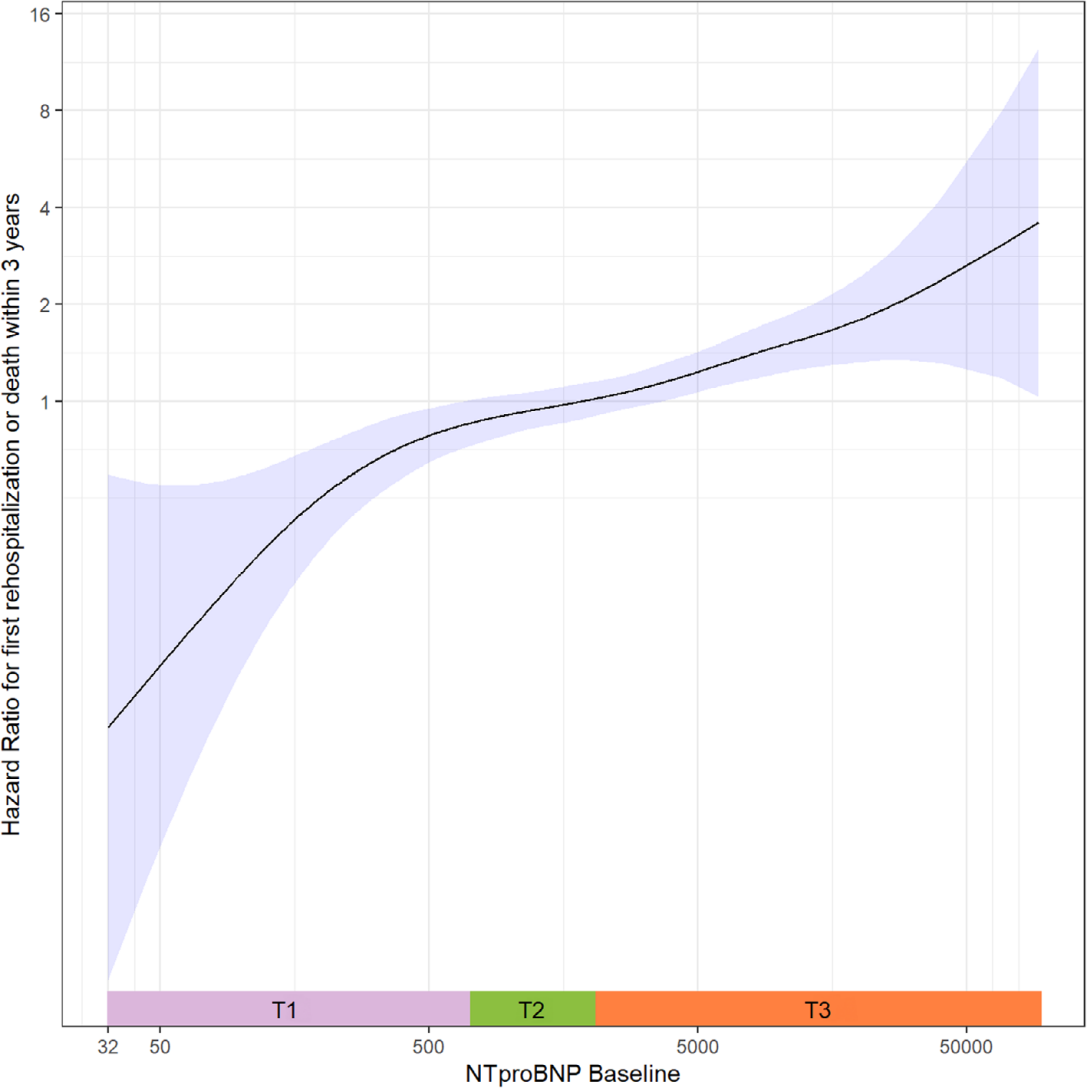
Unadjusted spline curve depicting the association between pre‐interventional N‐terminal pro‐B‐type natriuretic peptide (NT‐proBNP) levels and hazard ratio for all‐cause mortality or heart failure hospitalization within 3 years. NT‐proBNP values were logarithmically transformed, with the median set as the reference point (hazard ratio = 1). The risk increases progressively with higher NT‐proBNP levels, as shown by the steep rise in the hazard ratio, particularly at the upper end.

Variables with a *p* < 0.25 in univariable analysis were included in the multivariable model, while those with *p* ≥ 0.25 are listed in online supplementary *Table* *S5*. Multivariable Cox regression analysis showed log‐transformed NT‐proBNP as an independent predictor of the primary endpoint (adjusted hazard ratio [HR] 1.17, 95% CI 1.07–1.28; *p* < 0.001). Additional factors identified through backwards regression analysis included NYHA class, haemoglobin, creatinine, and atrial fibrillation (*Table* *4*). In the multivariable model, only NYHA class, haemoglobin level, and creatinine remained significant. An unadjusted spline curve depicting the association between NT‐proBNP and the primary endpoint is shown in *Figure* *4*. There was a continuous increase in the risk of the primary endpoint across the whole range of NT‐proBNP values.

**Table 4:** Cox proportional hazards model for the primary endpoint

Variable	Univariable analysis			Multivariable analysis		
	Hazard ratio	95% CI	*p*‐value	Hazard ratio	95% CI	*p*‐value
NT‐proBNP (log)	1.31	1.20–1.43	<0.001	1.17	1.07–1.28	**<0.001**
Age	1.01	1.00–1.03	0.120	–	–	–
NYHA class			<0.001			
I	Reference			–	–	–
II	0.42	0.14–1.20	0.100	0.58	0.21–1.62	0.300
III	1.39	0.36–2.70	0.980	0.90	0.33–2.43	0.840
IV	1.39	0.50–3.86	0.530	1.05	0.38–2.89	0.930
Six‐min walking distance	1.00	1.00–1.00	<0.001	^b^	^b^	^b^
MLHFQ	1.01	1.00–1.02	0.052	^b^	^b^	^b^
Haemoglobin	0.88	0.83–0.93	<0.001	0.91	0.86–0.97	**0.001**
Creatinine	1.33	1.18–1.49	<0.001	1.20	1.05–1.35	**0.005**
Urea	1.01	1.00–1.01	<0.001	^b^	^b^	^b^
Diuretics^a^			0.038	–	–	–
No diuretics	Reference			–	–	–
One agent	1.48	1.07–2.04	0.018	–	–	–
Two agents	1.57	1.01–2.45	0.047	–	–	–
Diabetes mellitus	1.38	1.07–1.78	0.014	–	–	–
Atrial fibrillation	1.42	1.12–1.79	0.004	1.13	0.90–1.41	0.300
Chronic lung disease	1.22	0.95–1.56	0.130	–	–	–
Peripheral artery disease	1.35	1.00–1.81	0.048	–	–	–
EuroSCORE II	1.05	1.02–1.08	<0.001	^b^	^b^	^b^
STS‐PROM (MV repair)	1.06	1.02–1.09	<0.001	^b^	^b^	^b^
History of TAVR	1.41	0.89–2.24	0.140	–	–	–
History of hospitalization for heart failure 12 months prior	1.58	1.21–2.06	<0.001	^b^	^b^	^b^
Main jet direction			0.010	^b^	^b^	^b^
Centric	Reference			^b^	^b^	^b^
Eccentric anterior	0.68	0.50–0.94	0.018	^b^	^b^	^b^
Eccentric posterior	1.00	0.77–1.42	0.800	^b^	^b^	^b^
Flail width	0.96	0.95–1.02	0.200	^b^	^b^	^b^
Mitral annular calcification			0.001	^b^	^b^	^b^
None	Reference			–	–	–
Spots	2.03	1.06–3.90	0.033	–	–	–
Anterior ring	0.30	0.04–2.21	0.240	–	–	–
Posterior ring	0.60	0.32–1.15	0.120	–	–	–
Total annulus	0.68	0.24–1.87	0.450	–	–	–
Annulus including leaflets	2.73	1.48–5.05	0.001	–	–	–
Anterior mitral leaflet length	1.02	0.99–1.05	0.160	^b^	^b^	^b^
TAPSE/PASP ratio	0.40	0.21–0.75	0.004	^b^	^b^	^b^
Tricuspid regurgitation severity			<0.001	–	–	–
None/trace	Reference			–	–	–
Mild	1.12	0.64–1.96	0.680	–	–	–
Moderate	1.40	0.80–2.45	0.240	–	–	–
Severe	2.00	1.12–3.56	0.019	–	–	–
History of cardiac surgery	1.17	0.91–1.51	0.230	–	–	–
Left atrial volume index	1.00	1.00–1.01	0.070	^b^	^b^	^b^

CI confidence interval; EuroSCORE, European System for Cardiac Operative Risk Evaluation; MLHFQ, Minnesota Living with Heart Failure Questionnaire; MV, mitral valve; NT‐proBNP, N‐terminal pro‐B‐type natriuretic peptide; NYHA, New York Heart Association; PASP, pulmonary artery systolic pressure; STS‐PROM, Society of Thoracic Surgeons predicted risk of mortality; TAPSE, tricuspid annular plane systolic excursion; TAVR, transcatheter aortic valve replacement.

^a^ Excluding mineralocorticoid receptor antagonists.

^b^ Removed from multivariable analysis due to >20% missing values.

#### Sensitivity analyses for the multivariable Cox regression model

We performed sensitivity analyses for comorbidities that may interfere with NT‐proBNP clearance (chronic kidney disease, obesity), as well as in patients with isolated PMR after excluding mixed MR, and using a dichotomized NT‐proBNP threshold of 1000 ng/L. Testing the Cox proportional hazards model using corrected NT‐proBNP for renal function confirmed the independent association with the primary endpoint (adjusted HR 1.24, 95% CI 1.03–1.25; *p* = 0.013; online supplementary *Table* *S6*). This association was also confirmed when analysing 679 patients with isolated PMR (adjusted HR 1.27, 95% CI 1.07–1.43; *p* = 0.003; online supplementary *Table* *S7*). In 130 obese patients, however, this association was not observed (adjusted HR 1.03, 95% CI 0.74–1.45; *p* = 0.850; online supplementary *Table* *S8*). A sensitivity analysis using a dichotomized NT‐proBNP threshold of 1000 ng/L showed an association with the primary endpoint in univariable analysis (HR 1.73, 95% CI 1.33–2.24; *p* < 0.001) but narrowly not in the multivariable model, although with the same trend (HR 1.30, 95% CI 1.00–1.69; *p* = 0.053; online supplementary *Table* *S9*).

### mMIDA score analysis

In the mMIDA score analysis, 144 patients with three or more missing parameters and 197 patients from centres lacking mMIDA measurements were excluded, resulting in 1041 patients. The mMIDA score, including imputed variables, is presented in *Table* *5*, with missing values shown in online supplementary *Table* *S10*. Median mMIDA scores across NT‐proBNP tertiles were lowest in T1 (8.0 points [6.0–9.0]) and higher in T2 (9.0 points [7.8–10.0]), and T3 (9.2 points [8.0–10.6]; *p* < 0.001 across groups).

**Table 5:** Contributors to mMIDA score according to NT‐proBNP tertiles for patients included in the mMIDA analysis^a^

	All (*n* = 1382)	1^st^ Tertile (*n* = 461)	2^nd^ Tertile (*n* = 459)	3^rd^ Tertile (*n* = 462)	*p*‐value
Age ≥65 years	985 (94.62)	319 (93.27)	329 (94.27)	337 (96.29)	0.200
NYHA class ≥III	876 (84.16)	258 (75.31)	297 (85.07)	322 (91.90)	**<0.001**
Atrial fibrillation	702 (67.40)	173 (50.47)	262 (75.07)	267 (76.29)	**<0.001**
LAVi ≥60 ml/m^2^	618 (59.41)	166 (48.50)	226 (64.77)	227 (64.73)	**<0.001**
PASP ≥50 mmHg	507 (48.7)	121 (35.4)	180 (51.5)	206 (59.0)	**<0.001**
LVESD ≥40 mm	379 (36.37)	106 (30.87)	126 (36.14)	147 (41.99)	**0.016**
LVEF <60%	568 (54.61)	152 (44.43)	191 (54.69)	226 (64.47)	**<0.001**
mMIDA	9.0 (7.0–10.0)	8.0 (6.0–9.0)	9.0 (7.8–10.0)	9.2 (8.0–10.6)	**<0.001**

Values are *n* (%), or median (Q1–Q3).

LAVi, left atrial volume index; LVEF, left ventricular ejection fraction; LVESD, left ventricular end‐systolic diameter; mMIDA, modified Mitral Regurgitation International Database; NT‐proBNP, N‐terminal pro‐B‐type natriuretic peptide; NYHA, New York Heart Association; PASP, pulmonary artery systolic pressure.

^a^ Missing variables are estimated.

Of the 1041 patients in the mMIDA analysis, 301 reached the primary endpoint. In univariable Cox regression, the continuous mMIDA score was associated with the primary endpoint (HR 1.10, 95% CI 1.04–1.17; *p* = 0.002) per one score point. After adjusting for log‐transformed NT‐proBNP, the mMIDA score association was no longer significant (HR 1.06, 95% CI 0.99–1.13; *p* = 0.083), while NT‐proBNP remained significantly associated (HR 1.20, 95% CI 1.07–1.34; *p* = 0.002).

## Discussion

In this large, international, multicentre study, we investigated the prognostic value of pre‐interventional NT‐proBNP levels in PMR patients undergoing M‐TEER. To the best of our knowledge, this is the largest study to date to address this question. The main findings can be summarized as follows: (i) NT‐proBNP levels were independently and quantitatively associated with the composite endpoint of all‐cause mortality or HF hospitalization within 3 years; (ii) while the mMIDA score initially showed an association with the primary endpoint, this association lost significance after adjusting for NT‐proBNP; and (iii) real‐world PMR patients undergoing M‐TEER represent a heterogeneous population, as reflected by the wide range of NT‐proBNP levels, with continuous risk increase across the whole range of NT‐proBNP levels (*Graphical Abstract*).

### Need for outcome prediction in primary mitral regurgitation patients undergoing transcatheter edge‐to‐edge repair

Dedicated outcome prediction in PMR patients undergoing M‐TEER is currently lacking. Despite the increasing number of PMR patients being treated with M‐TEER, there is a paucity of data on pre‐interventional outcome prediction models tailored specifically for this population and existing prognostic assessments are limited to smaller analyses.^12^, ^13^, ^14^, ^15^, ^16^ Therefore, there is an essential need for prognostic tools to guide clinical decision‐making in PMR patients undergoing M‐TEER.

### Prognostic value of NT‐proBNP

Consistent with our hypothesis, NT‐proBNP levels were independently associated with the primary endpoint of all‐cause death or HF hospitalization within 3 years. Even after adjusting for various echocardiographic and clinical parameters, NT‐proBNP remained significantly associated with adverse outcomes, underscoring its value as a prognostic biomarker in PMR patients. The robustness of this association was confirmed in multiple sensitivity analyses. Adjusting for renal function and focusing on patients with isolated PMR (vs. mixed MR) still demonstrated a significant and independent association with the primary endpoint. The only exception was in obese patients (BMI >30 kg/m^2^), where the association was lost—a known limitation of natriuretic peptides, potentially due to reduced B‐type natriuretic peptide [BNP] production, altered peripheral adipose tissue metabolism, and impaired processing of proBNP, leading to decreased plasma BNP levels in obese individuals.^17^, ^18^ These findings demonstrate NT‐proBNP's prognostic utility and applicability to a broad PMR population.

Exploring serial measurements of NT‐proBNP after intervention in PMR patients could be valuable for future research, as significant reductions have been associated with favourable prognostic outcomes in other patient populations.^19^ Monitoring NT‐proBNP dynamics following M‐TEER may help identify patients with isolated valvular HF who might benefit most from the procedure, since baseline NT‐proBNP levels may not fully capture the prognostic benefit in these cases, given that M‐TEER addresses the valvular component.

Despite similar baseline MR grade across NT‐proBNP tertiles, patients with lower NT‐proBNP levels more frequently achieved MR reduction to ≤1+. This might be due to the higher prevalence of mitral annular and MV leaflet calcification in the highest NT‐proBNP tertile, potentially resulting from an interplay between renal dysfunction (leading to elevated NT‐proBNP levels) and calcification processes.^20^ Increased calcification can lead to more challenging M‐TEER procedures and worse outcomes.^21^, ^22^ The relationship between NT‐proBNP levels and reduced MR reduction following M‐TEER in PMR patients warrants further investigation.

### Prognostic value of the MIDA score

The broad range of pathophysiologically relevant parameters included in the MIDA score, as well as its validation in PMR patients undergoing MV surgery, render it potentially powerful as an outcome predictor in PMR patients undergoing M‐TEER.^6^, ^7^ Considering that PMR patients referred for M‐TEER are typically symptomatic and that LA diameter is not anymore routinely measured in clinical practice, we modified the MIDA score according to the more recently published MIDA‐Q score, adjusting the NYHA class from ≥II to ≥III and replacing the LA diameter of ≥55 mm with an LA volume index of ≥60 ml/m^2^.^9^ However, it is important to note that the applied mMIDA score in this form has not been validated.

Although the mMIDA score was associated with the primary endpoint in univariable analysis, it lost significance after adjusting for NT‐proBNP, which remained a strong and independent predictor. The association of NT‐proBNP with parameters of the mMIDA score—such as advanced age, symptomatic burden, prevalence of atrial fibrillation, atrial and/or ventricular dysfunction, and right ventricular overload—suggests that NT‐proBNP may provide a more comprehensive, objective, and easily accessible risk assessment, independent of echocardiographic expertise. Importantly, NT‐proBNP seems to be more reliable as prognostic predictor than the multiparametric and mMIDA score in patients with PMR undergoing M‐TEER.

### Real‐world primary mitral regurgitation patients undergoing transcatheter edge‐to‐edge repair represent a heterogeneous population

Our analysis by NT‐proBNP tertiles revealed significant heterogeneity among PMR patients undergoing M‐TEER. Higher NT‐proBNP tertiles included older patients with elevated surgical risk scores, a higher prevalence of comorbidities (e.g. atrial fibrillation, coronary artery disease, prior myocardial infarction, renal dysfunction), greater symptomatic burden (including a history of HF hospitalizations), and more advanced echocardiographic signs of left and right heart congestion. Notably, MR severity did not differ significantly across tertiles, aligning with previous analyses and emphasizing that natriuretic peptide activation reflects haemodynamic consequences, rather than MR severity itself.^23^

These findings reinforce real‐world data suggesting delayed referral for MV intervention and demonstrate that severe PMR often extends beyond isolated MV disease, encompassing a spectrum of HF symptoms due to volume overload from untreated MR.^24^ This is further reflected by the high rates of all‐cause mortality and HF hospitalizations in our population, exceeding those observed in contemporary and historical PMR M‐TEER studies, which likely reflects the inclusion of a real‐world PMR population without restrictive inclusion criteria, covering the full PMR spectrum.^25^, ^26^

### Considerations

Two important aspects should be considered. First, a biomarker like NT‐proBNP should always complement, not replace, thorough clinical and echocardiographic risk evaluation, and should be interpreted within the context of individual patient characteristics and comorbidities. Moreover, we deliberately chose not to report a fixed optimized NT‐proBNP cut‐off for risk stratification associated with the primary endpoint, as this study focuses on symptomatic patients selected for M‐TEER and the risk of reaching the primary endpoint increases continuously with higher NT‐proBNP levels, as illustrated by the spline analysis; therefore, applying a rigid cut‐off could oversimplify its prognostic value. Second, while it seems clinically reasonable that patients with elevated NT‐proBNP and suspected PMR should undergo timely and detailed clinical as well as echocardiographic evaluation, our findings cannot establish a threshold for intervention. Determining an intervention threshold warrants further investigation in prospective studies, especially among asymptomatic patients who may qualify for M‐TEER.

### Study limitations

This study has limitations inherent to its retrospective, observational design. First, NT‐proBNP levels and survival status at follow‐up were available in 1382 patients of the PRIME‐MR registry, potentially introducing selection bias. A comparative analysis of included and excluded patients (online supplementary *Table Appendix* *S1*) showed that excluded patients were slightly older, had a higher prevalence of coronary artery disease and atrial fibrillation, and were more frequently in NYHA class IV. They also had a higher rate of prior HF hospitalizations, whereas echocardiographic parameters were more advanced in included patients. These differences suggest that missing NT‐proBNP levels and survival data were not entirely random, raising the possibility that NT‐proBNP testing was performed preferentially in certain clinical scenarios. Second, while the adaptation of the original MIDA score by replacing LA diameter ≥55 mm with LA volume index ≥60 ml/m^2^ and NYHA class ≥II with ≥III is supported by the MIDA‐Q score, it is important to emphasize that the mMIDA score itself has not been validated.^9^ Of note, analysing isolated MIDA score parameters of the modified (online supplementary *Tables* *S11* and *S12*) and original (online supplementary *Tables* *S13* and *S14*) MIDA score supports the use of NYHA class ≥III over ≥II in our study, as this was independently associated with our primary endpoint. However, LA diameter was not obtained in our study. Third, the study covers a long period (2008–2022), during which advances in M‐TEER technology and changes in patient selection criteria may affect generalizability. Fourth, while efforts were made to impute missing values, incomplete data could still affect results. Fifth, as an observational study, our analysis is inherently limited in its ability to establish causality. While we identified a strong association between NT‐proBNP and clinical outcomes, the findings should be interpreted as hypothesis‐generating. Prospective studies are needed to validate these results and determine their implications for clinical decision‐making. Lastly, ethnicity data were not collected, which may be relevant as natriuretic peptide levels can vary by ethnic background, with higher levels observed in Asian and African descent populations compared to Caucasian and Hispanic populations.^27^

## Conclusions

NT‐proBNP is a valuable prognostic marker in PMR patients undergoing M‐TEER. Higher pre‐interventional NT‐proBNP levels are independently associated with increased all‐cause mortality or HF hospitalizations, despite treatment. These findings suggest that incorporating NT‐proBNP into clinical assessment may improve risk stratification and potentially advocates for earlier intervention at lower NT‐proBNP levels to optimize outcomes in PMR patients undergoing M‐TEER.

## Supporting information


**Appendix S1.** Supporting Information.
