# Supplementary material for: Prognostic value of NT‐proBNP in patients with primary mitral regurgitation undergoing transcatheter edge‐to‐edge repair
Source: Eur J Heart Fail. 2025 Jun 18;27(12):2921–34. doi: 10.1002/ejhf.3725 (PMC12803553; doi:10.1002/ejhf.3725)
Supplement: Supplementary file 1 — Appendix S1. Supporting Information. [file EJHF-27-2921-s001.docx]

# SUPPLEMENTAL MATERIAL

## Supplemental Table 1. Clinical Baseline Characteristics of Included and Excluded Patients

|  | **All**  **(N=3,083)** | **Included**  **(N=1,382)** | **Excluded**  **(N=1,701)** | **P value** |
| --- | --- | --- | --- | --- |
| **Demographic** |  |  |  |  |
| Age, years | 82.0 (76.0, 85.0) | 81.0 (76.0, 85.0) | 82.0 (76.0, 86.0) | **<0.001** |
| Male | 1690 (54.9) | 727 (52.6) | 963 (56.6) | **0.029** |
| BMI, kg/m² | 24.4 (21.8, 27.7) | 24.7 (22.1, 27.7) | 24.3 (21.5, 27.5) | **0.026** |
| **Surgical Risk Scores** |  |  |  |  |
| EuroSCORE II, % | 4.1 (2.5, 6.6) | 4.1 (2.4, 6.9) | 4.1 (2.5, 6.4) | 0.980 |
| STS-PROM (MV repair), % | 4.0 (2.5, 6.6) | 3.8 (2.4, 6.2) | 4.1 (2.5, 6.8) | 0.050 |
| **Comorbidities** |  |  |  |  |
| Arterial hypertension | 2327 (77.1) | 1047 (76.4) | 1280 (77.7) | 0.420 |
| Diabetes mellitus | 504 (16.9) | 220 (16.1) | 284 (17.6) | 0.280 |
| Coronary artery disease | 1067 (41.0) | 593 (43.4) | 474 (38.3) | **0.001** |
| Atrial fibrillation | 1955 (64.0) | 926 (67.1) | 1029 (61.5) | **0.002** |
| Chronic lung disease | 484 (16.0) | 229 (16.6) | 255 (15.5) | 0.460 |
| History of stroke | 302 (10.0) | 153 (11.1) | 149 (9.1) | 0.072 |
| History of myocardial infarction | 376 (12.7) | 201 (15.0) | 175 (10.8) | **<0.001** |
| History of cardiac surgery | 552 (17.9) | 231 (16.7) | 321 (18.9) | 0.130 |
| **Clinical Presentation** |  |  |  |  |
| NYHA class |  |  |  |  |
| I | 27 (0.9) | 12 (0.9) | 15 (0.9) | >0.999 |
| II | 565 (18.7) | 231 (17.0) | 334 (20.1) | **0.032** |
| III | 1786 (59.2) | 904 (66.5) | 882 (53.2) | **<0.001** |
| IV | 640 (21.2) | 212 (15.6) | 428 (25.8) | **<0.001** |
| Six-minute walking distance, m | 220.0 (121.5, 320.0) | 215.0 (120.0, 315.5) | 240.0 (146.5, 360.0) | 0.084 |
| MLHFQ, points | 34.0 (23.0, 45.0) | 34.0 (22.0, 46.0) | 33.0 (24.0, 44.0) | 0.940 |
| History of hospitalisation for heart failure 12 months prior | 1221 (54.5) | 388 (43.3) | 833 (62.0) | **<0.001** |
| **Laboratory Results** |  |  |  |  |
| Haemoglobin (g/dL) | 12.3 (11.0, 13.5) | 12.3 (10.9, 13.5) | 12.3 (11.0, 13.5) | 0.440 |
| NT-proBNP (pg/mL) | 2038.0 (847.5, 4294.8) | 1991.0 (828.8, 4260.0) | 2283.0 (1003.5, 4644.8) | 0.280 |
| Renal function |  |  |  |  |
| Creatinine, mg/dL | 1.2 (0.9, 1.5) | 1.2 (0.9, 1.5) | 1.2 (0.9, 1.5) | 0.970 |
| eGFR, mL/min | 50.1 (36.0, 65.6) | 49.1 (36.0, 65.6) | 50.7 (36.1, 65.5) | 0.480 |
| On dialysis | 53 (1.8) | 20 (1.5) | 33 (2.0) | 0.280 |
|  | | | | |
| Values are n (%), median (Q1, Q3), or mean ± SD. **Bold** values indicate statistical significance. *BMI = body mass index; eGFR = estimated glomerular filtration rate; EuroSCORE = European System for Cardiac Operative Risk Evaluation; mMIDA = modified Mitral Regurgitation International Database; MLHFQ = Minnesota living with heart failure questionnaire; MV = mitral valve; NYHA = New York Heart Association; NT-proBNP = N-terminal pro-B-type natriuretic peptide; STS- PROM = Society of Thoracic Surgeons Predicted Risk of Mortality* | | | | |

## Supplemental Table 2. Echocardiographic Baseline Characteristics of Included and Excluded Patients

|  | **All**  **(N=3,083)** | **Included**  **(N=1,382)** | **Excluded**  **(N=1,701)** | **P value** |
| --- | --- | --- | --- | --- |
| **Left Atrium** |  |  |  |  |
| Left atrial volume, mL | 107.0 (80.0, 141.0) | 111.0 (80.0, 146.0) | 104.5 (80.0, 136.0) | **0.001** |
| Left atrial volume index, mL/m^2^ | 63.0 (46.0, 82.0) | 66.0 (48.0, 85.0) | 60.0 (45.0, 79.0) | **<0.001** |
| **Left Ventricle** |  |  |  |  |
| LVEF, % | 60.0 (51.0, 65.0) | 58.0 (50.0, 64.0) | 60.0 (51.0, 65.0) | **<0.001** |
| LVEDD, mm | 52.0 (47.0, 58.0) | 53.0 (47.0, 59.0) | 52.0 (47.0, 58.0) | **0.044** |
| LVESD, mm | 35.0 (29.0, 42.0) | 36.0 (30.0, 43.0) | 34.0 (28.0, 40.0) | **<0.001** |
| LVEDV, mL | 110.0 (83.0, 143.0) | 112.0 (86.0, 145.0) | 107.0 (80.0, 140.0) | **0.006** |
| LVESV, mL | 45.0 (31.0, 64.0) | 46.0 (33.9, 66.0) | 44.0 (30.0, 63.0) | **0.004** |
| **Mitral Valve** |  |  |  |  |
| Mean transmitral gradient, mmHg | 2.3 (2.0, 3.2) | 2.0 (2.0, 3.0) | 2.9 (2.0, 4.0) | **0.003** |
| 3D MVOA, cm^2^ | 4.5 (3.8, 5.6) | 4.4 (3.6, 5.6) | 4.7 (4.0, 5.7) | 0.180 |
| AML length, mm | 24.0 (20.7, 28.0) | 23.0 (20.0, 27.0) | 25.0 (21.5, 28.6) | **<0.001** |
| PML length, mm | 13.5 (11.0, 16.0) | 13.0 (11.0, 16.0) | 14.0 (12.0, 17.0) | **0.012** |
| Mitral annular calcification in 3D | 1283 (73.9) | 437 (75.0) | 846 (73.4) | 0.530 |
| Anterior ring | 22 (1.3) | 13 (2.2) | 9 (0.8) | **0.020** |
| Posterior ring | 260 (15.0) | 53 (9.1) | 207 (18.0) | **<0.001** |
| Total annulus | 36 (2.1) | 22 (3.8) | 14 (1.2) | **<0.001** |
| Annulus including leaflets | 53 (3.1) | 31 (5.3) | 22 (1.9) | **<0.001** |
| **Mitral Regurgitation** |  |  |  |  |
| EROA, cm^2^ | 0.4 (0.3, 0.6) | 0.4 (0.3, 0.6) | 0.4 (0.2, 0.6) | **0.005** |
| Regurgitant volume, mL | 65.5 (49.0, 85.0) | 64.0 (50.0, 82.6) | 66.5 (47.8, 86.5) | 0.590 |
| Grade 3+ | 591 (20.4) | 315 (24.0) | 276 (17.4) | **<0.001** |
| Grade 4+ | 2310 (79.6) | 999 (76.0) | 1311 (82.6) | **<0.001** |
| Aetiology |  |  |  |  |
| Isolated PMR | 1704 (81.8) | 828 (81.7) | 876 (81.8) | >0.999 |
| Mixed, leading cause PMR | 380 (18.2) | 185 (18.3) | 195 (18.2) | >0.999 |
| Main pathology |  |  |  |  |
| Flail | 1170 (44.1) | 526 (47.4) | 644 (41.7) | **0.004** |
| Flail gap, mm | 5.0 (3.4, 7.0) | 5.0 (3.0, 7.0) | 5.0 (4.0, 7.0) | **0.009** |
| Flail width, mm | 11.0 (8.0, 14.0) | 10.0 (7.0, 14.0) | 11.0 (8.0, 15.0) | **0.006** |
| Prolapse | 1331 (50.2) | 503 (45.3) | 828 (53.6) | **<0.001** |
| Calcification | 111 (4.2) | 53 (4.8) | 58 (3.8) | 0.230 |
| Segment of main pathology |  |  |  |  |
| A1 | 48 (2.6) | 33 (3.6) | 15 (1.7) | **0.015** |
| A2 | 511 (28.0) | 269 (29.3) | 242 (26.7) | 0.240 |
| A3 | 58 (3.2) | 37 (4.0) | 21 (2.3) | 0.051 |
| P1 | 131 (7.2) | 67 (7.3) | 64 (7.1) | 0.920 |
| P2 | 907 (49.7) | 435 (47.4) | 472 (52.1) | **0.049** |
| P3 | 139 (7.6) | 69 (7.5) | 70 (7.7) | 0.940 |
| **Right Ventricle** |  |  |  |  |
| Severe tricuspid regurgitation | 562 (19.9) | 277 (22.1) | 285 (18.2) | **0.012** |
| TAPSE, mm | 19.0 (16.0, 23.0) | 19.0 (16.0, 23.0) | 20.0 (16.0, 23.0) | **0.029** |
| PASP, mmHg | 47.0 (36.0, 60.0) | 48.0 (37.0, 60.0) | 47.0 (36.0, 58.1) | 0.210 |
| TAPSE/PASP ratio, mm/mmHg | 0.4 (0.3, 0.5) | 0.4 (0.3, 0.6) | 0.4 (0.3, 0.5) | 0.950 |
|  |  |  |  |  |
| Values are n (%) or median (Q1, Q3). **Bold** values indicate statistical significance. *AML = anterior mitral leaflet;* *EROA = effective regurgitant orifice area; LVEF = left ventricular ejection fraction; LVEDD = left ventricular end-diastolic diameter; LVEDV = left ventricular end-diastolic volume; LVESD = left ventricular end-systolic diameter; LVESV = left ventricular end-systolic volume; MVOA = mitral valve orifice area; PML = posterior mitral leaflet; PMR = primary mitral regurgitation* | | | | |

## Supplemental Table 3. Baseline Medication According to NT-proBNP Tertiles

| **Medication** | **All**  **(N=1,382)** | **1^st^ Tertile**  **(N=461)** | **2^nd^ Tertile**  **(N=459)** | **3^rd^ Tertile**  **(N=462)** | **P value** |
| --- | --- | --- | --- | --- | --- |
| Angiotensin-converting-enzyme inhibitors | 559 (41.4) | 203 (45.5) | 174 (38.4) | 182 (40.3) | 0.081 |
| Angiotensin II receptor blockers | 257 (19.0) | 86 (19.3) | 84 (18.5) | 87 (19.2) | 0.95 |
| Angiotensin receptor-neprilysin inhibitor | 30 (2.2) | 11 (2.5) | 8 (1.8) | 11 (2.4) | 0.72 |
| Betablocker | 951 (69.8) | 256 (56.8) | 338 (74.3) | 357 (78.3) | **<0.001** |
| Mineralocorticoid receptor antagonist | 303 (24.9) | 75 (19.2) | 110 (26.6) | 118 (28.6) | **0.005** |
| Diuretics* | 1060 (84.1) | 357 (75.3) | 399 (85.9) | 426 (91.3) | **<0.001** |
| No diuretics | 200 (15.9) | 104 (24.7) | 60 (14.1) | 36 (8.7) | **<0.001** |
| One agent | 957 (76.0) | 298 (70.8) | 330 (77.3) | 329 (79.9) | **0.007** |
| Two agents | 103 (8.2) | 19 (4.5) | 37 (8.7) | 47 (11.4) | **0.001** |
| Loop diuretic daily dose, mg | 40 (20, 80) | 40 (0, 60) | 40 (20, 80) | 60 (40, 100) | **<0.001** |
|  | | | | | |
| Values are n (%) or median (Q1, Q3). **Bold** values indicate statistical significance. *Excluding mineralocorticoid receptor antagonists. | | | | | |

## Supplemental Table 4. Device Selection and Device Generation According to NT-proBNP Tertiles

| **Device** | **All**  **(N=1,382)** | **1^st^ Tertile**  **(N=461)** | **2^nd^ Tertile**  **(N=459)** | **3^rd^ Tertile**  **(N=462)** | **P value** |
| --- | --- | --- | --- | --- | --- |
| MitraClip G1 (%) |  |  |  |  |  |
| 1^st^ device | 494 (41.6) | 159 (38.6) | 163 (41.2) | 172 (45.3) | 0.160 |
| 2^nd^ device | 227 (41.8) | 63 (34.1) | 70 (40.9) | 94 (50.3) | **0.006** |
| 3^rd^ device | 16 (27.1) | 6 (27.3) | 4 (23.5) | 6 (30.0) | 0.910 |
| MitraClip G2 (%) |  |  |  |  |  |
| 1^st^ device | 120 (10.1) | 47 (11.4) | 36 (9.1) | 37 (9.7) | 0.530 |
| 2^nd^ device | 42 (7.7) | 16 (8.6) | 15 (8.8) | 11 (5.9) | 0.500 |
| 3^rd^ device | 9 (15.3) | 3 (13.6) | 2 (11.8) | 4 (20.0) | 0.760 |
| MitraClip G3 XTR (%) |  |  |  |  |  |
| 1^st^ device | 198 (16.7) | 85 (20.6) | 61 (15.4) | 52 (13.7) | **0.023** |
| 2^nd^ device | 91 (16.8) | 37 (20.0) | 28 (16.4) | 26 (13.9) | 0.290 |
| 3^rd^ device | 10 (16.9) | 1 (4.5) | 5 (29.4) | 4 (20.0) | 0.110 |
| MitraClip G3 NTR (%) |  |  |  |  |  |
| 1^st^ device | 147 (12.4) | 48 (11.7) | 46 (11.6) | 53 (13.9) | 0.530 |
| 2^nd^ device | 94 (17.3) | 33 (17.8) | 30 (17.5) | 31 (16.6) | 0.950 |
| 3^rd^ device | 12 (20.3) | 7 (31.8) | 1 (5.9) | 4 (20.0) | 0.140 |
| MitraClip G4 XT (%) |  |  |  |  |  |
| 1^st^ device | 18 (1.5) | 9 (2.2) | 6 (1.5) | 3 (0.8) | 0.280 |
| 2^nd^ device | 16 (2.9) | 11 (5.9) | 3 (1.8) | 2 (1.1) | **0.011** |
| 3^rd^ device | 3 (5.1) | 1 (4.5) | 0 (0) | 2 (10.0) | 0.380 |
| MitraClip G4 XTW (%) |  |  |  |  |  |
| 1^st^ device | 49 (4.1) | 16 (3.9) | 25 (6.3) | 8 (2.1) | **0.012** |
| 2^nd^ device | 11 (2.0) | 3 (1.6) | 6 (3.5) | 2 (1.1) | 0.230 |
| 3^rd^ device | 0 (0) | 0 (0) | 0 (0) | 0 (0) |  |
| MitraClip G4 NT (%) |  |  |  |  |  |
| 1^st^ device | 22 (1.9) | 13 (3.2) | 7 (1.8) | 2 (0.5) | **0.023** |
| 2^nd^ device | 17 (3.1) | 8 (4.3) | 5 (2.9) | 4 (2.1) | 0.470 |
| 3^rd^ device | 6 (10.2) | 2 (9.1) | 4 (23.5) | 0 (0) | 0.060 |
| MitraClip G4 NTW (%) |  |  |  |  |  |
| 1^st^ device | 24 (2.0) | 7 (1.7) | 11 (2.8) | 6 (1.6) | 0.420 |
| 2^nd^ device | 2 (0.4) | 0 (0) | 1 (0.6) | 1 (0.5) | 0.590 |
| 3^rd^ device | 0 (0) | 0 (0) | 0 (0) | 0 (0) |  |
| PASCAL P10 (%) |  |  |  |  |  |
| 1^st^ device | 49 (4.1) | 12 (2.9) | 14 (3.5) | 23 (6.1) | 0.066 |
| 2^nd^ device | 10 (1.8) | 5 (2.7) | 1 (0.6) | 4 (2.1) | 0.310 |
| 3^rd^ device | 0 (0) | 0 (0) | 0 (0) | 0 (0) |  |
| PASCAL Ace (%) |  |  |  |  |  |
| 1^st^ device | 67 (5.6) | 16 (3.9) | 27 (6.8) | 24 (6.3) | 0.150 |
| 2^nd^ device | 33 (6.1) | 9 (4.9) | 12 (7.0) | 12 (6.4) | 0.680 |
| 3^rd^ device | 3 (5.1) | 2 (9.1) | 1 (5.9) | 0 (0) | 0.400 |
|  | | | | | |
| Values are n (%). **Bold** values indicate statistical significance. | | | | | |

## Supplemental Table 5. Cox Proportional Hazards Model for the Primary Endpoint – Non-significant Parameters

| **Variable** | **Univariable Analysis** | | |
| --- | --- | --- | --- |
|  | **Hazard Ratio** | **95% Confidence Interval** | **P value** |
| Male | 1.09 | (0.89, 1.34) | 0.410 |
| Renin–angiotensin–aldosterone system inhibitors |  |  | 0.720 |
| None | Reference |  |  |
| Angiotensin-converting-enzyme inhibitors | 0.90 | (0.71, 1.15) | 0.390 |
| Angiotensin II receptor blockers | 0.88 | (0.65, 1.19) | 0.420 |
| Angiotensin receptor-neprilysin inhibitor | 1.25 | (0.53, 2.94) | 0.610 |
| Betablocker | 1.06 | (0.85, 1.33) | 0.600 |
| Mineralocorticoid receptor antagonists | 1.08 | (0.83, 1.40) | 0.570 |
| Arterial hypertension | 1.01 | (0.78, 1.29) | 0.960 |
| Coronary artery disease | 1.05 | (0.86, 1.30) | 0.620 |
| History of myocardial infarction | 1.10 | (0.84, 1.46) | 0.480 |
| History of stroke | 0.94 | (0.67, 1.32) | 0.730 |
| History of coronary artery bypass grafting | 1.10 | 0.82, 1.48 | 0.540 |
| History of atrioventricular valve surgery | 1.03 | 0.63, 1.66 | 0.920 |
| Mitral regurgitation severity |  |  |  |
| 3+ | Reference |  |  |
| 4+ | 0.94 | 0.72, 1.21 | 0.630 |
| Mitral regurgitation aetiology |  |  |  |
| Isolated PMR | Reference |  |  |
| Mixed mitral regurgitation, leading cause PMR | 1.17 | 0.84, 1.62 | 0.350 |
| Mean transmitral gradient | 1.05 | 0.96, 1.13 | 0.280 |
| Flail gap | 1.02 | 0.95, 1.08 | 0.630 |
| Posterior mitral leaflet length | 1.01 | 0.97, 1.04 | 0.670 |
| Mitral valve orifice area | 0.99 | 0.86, 1.14 | 0.890 |
| Anterior-posterior diameter | 1.01. | 0.99, 1.03 | 0.330 |
| Bicommissural diameter | 0.99 | 0.97, 1.01 | 0.350 |
| Left ventricular end-diastolic volume | 1.00 | 1.00, 1.00 | 0.940 |
| Left ventricular end-systolic volume | 1.00 | 1.00, 1.00 | 0.720 |
| Left ventricular ejection fraction | 1.00 | 0.99, 1.01 | 0.970 |
| Left ventricular end-diastolic diameter | 1.00 | 0.98, 1.01 | 0.8550 |
| Left ventricular end-systolic diameter | 1.04 | 0.98, 1.01 | 0.260 |
| E/E’ | 1.01 | 0.98, 1.03 | 0.550 |
| Body mass index | 1.00 | 0.98, 1.02 | 0.980 |
| Body surface area | 1.08 | 0.67, 1.73 | 0.750 |
|  | | | |
| **Bold** values indicate statistical significance. *MLWHFQ = Minnesota Living with Heart Failure Questionnaire* | | | |

## Supplemental Table 6. Preselected Cox Proportional Hazards Model for the Primary Endpoint with NT-proBNP Corrected for Renal Function

| **Variable** | **Multivariable Analysis** | | |
| --- | --- | --- | --- |
|  | **Hazard Ratio** | **95% Confidence Interval** | **P value** |
| Corrected NT-proBNP (log) | 1.14 | 1.03, 1.25 | **0.013** |
| NYHA class |  |  |  |
| I | Reference |  |  |
| II | 0.37 | 0.13, 1.07 | 0.066 |
| III | 0.74 | 0.27, 2.03 | 0.550 |
| IV | 0.90 | 0.32, 2.53 | 0.840 |
| Haemoglobin | 0.91 | 0.86, 0.96 | **0.001** |
| Creatinine | 1.27 | 1.12, 1.45 | **<0.001** |
| Atrial fibrillation | 1.28 | 1.01, 1.64 | **0.045** |
|  | | | |
| **Bold** values indicate statistical significance. *NYHA = New York Heart Association; NT-proBNP = N-terminal pro-B-type natriuretic peptide* | | | |

## Supplemental Table 7. Preselected Cox Proportional Hazards Model for the Primary Endpoint in Patients with Isolated Primary Mitral Regurgitation

| **Variable** | **Multivariable Analysis** | | |
| --- | --- | --- | --- |
|  | **Hazard Ratio** | **95% Confidence Interval** | **P value** |
| NT-proBNP (log) | 1.24 | 1.07, 1.43 | **0.003** |
| NYHA class |  |  |  |
| I | Reference |  |  |
| II | 0.34 | 0.10, 1.19 | 0.091 |
| III | 0.77 | 0.24, 2.50 | 0.660 |
| IV | 0.76 | 0.23, 2.55 | 0.660 |
| Haemoglobin | 0.85 | 0.78, 0.9 | **<0.001** |
| Creatinine | 1.37 | 1.05, 1.77 | **0.018** |
| Atrial fibrillation | 1.32 | 0.95, 1.84 | 0.094 |
|  | | | |
| **Bold** values indicate statistical significance. *NYHA = New York Heart Association; NT-proBNP = N-terminal pro-B-type natriuretic peptide* | | | |

## Supplemental Table 8. Preselected Cox Proportional Hazards Model for the Primary Endpoint in Obese Patients

| **Variable** | **Multivariable Analysis** | | |
| --- | --- | --- | --- |
|  | **Hazard Ratio** | **95% Confidence Interval** | **P value** |
| NT-proBNP (log) | 1.03 | 0.74, 1.45 | 0.850 |
| NYHA class |  |  |  |
| I | (Reference) |  |  |
| II | 0.09 | 0.01, 0.81 | **0.032** |
| III | 0.48 | 0.22, 1.06 | 0.069 |
| IV | - | - | - |
| Haemoglobin | 0.96 | 0.79, 1.16 | 0.660 |
| Creatinine | 1.56 | 0.90, 2.68 | 0.110 |
| Atrial fibrillation | 0.90 | 0.46, 1.78 | 0.770 |
|  | | | |
| **Bold** values indicate statistical significance. *NYHA = New York Heart Association; NT-proBNP = N-terminal pro-B-type natriuretic peptide* | | | |

## Supplemental Table 9. Preselected Cox Proportional Hazards Model for the Primary Endpoint using a Dichotomized NT-proBNP Threshold of 1,000 ng/L

| **Variable** | **Multivariable Analysis** | | |
| --- | --- | --- | --- |
|  | **Hazard Ratio** | **95% Confidence Interval** | **P value** |
| NT-proBNP >1,000 ng/L | 1.30 | 1.00, 1.69 | 0.053 |
| NYHA class |  |  |  |
| I | (Reference) |  |  |
| II | 0.62 | 0.22, 1.73 | 0.360 |
| III | 0.99 | 0.37, 2.66 | 0.980 |
| IV | 1.19 | 0.43, 3.25 | 0.740 |
| Haemoglobin | 0.90 | 0.86, 0.96 | **<0.001** |
| Creatinine | 1.23 | 1.09, 1.38 | **0.001** |
| Atrial fibrillation | 1.15 | 0.91, 1.41 | 0.240 |
|  | | | |
| **Bold** values indicate statistical significance. *NYHA = New York Heart Association; NT-proBNP = N-terminal pro-B-type natriuretic peptide* | | | |

## Supplemental Table 10. Missing Values for Contributors to MIDA Score According to NT-proBNP Quartiles for Patients Included in the MIDA Analysis

| **Medication** | **All**  **(N=1,041)** | **1^st^ Tertile**  **(N=342)** | **2^nd^ Tertile**  **(N=349)** | **3^rd^ Tertile**  **(N=350)** |
| --- | --- | --- | --- | --- |
| Age ≥65 years | - | - | - | - |
| NYHA class ≥III | 7 (0.7) | 4 (1.2) | 1 (0.3) | 2 (0.6) |
| Atrial fibrillation | 1 (0.1) | 1 (0.3) | - | - |
| LAVi ≥60 mL/m^2^ | 142 (13.6) | 46 (13.5) | 47 (13.5) | 49 (14.0) |
| PASP ≥50 mmHg | 109 (10.5) | 43 (12.6) | 26 (7.4) | 40 (11.4) |
| LVESD ≥40 mm | 186 (17.9) | 60 (17.5) | 58 (16.6) | 68 (19.4) |
| LVEF <60% | 170 (16.3) | 62 (18.1) | 52 (14.9) | 56 (16.0) |
|  | | | | |
| Values are n (%). **Bold** values indicate statistical significance. *LAVi = left atrial volume index; LVEF = left ventricular ejection fraction; LVESD = left ventricular end-systolic diameter MIDA: Mitral Regurgitation International Database; NYHA = New York Heart Association; PASP = pulmonary artery systolic pressure* | | | | |

## Supplemental Table 11. Cox Proportional Hazards Model for the Primary Endpoint Using Isolated Modified MIDA Score Parameters

| **Variable** | **Multivariable Analysis** | | |
| --- | --- | --- | --- |
|  | **Hazard Ratio** | **95% Confidence Interval** | **P value** |
| Age ≥65 years | 1.07 | 0.90, 1.15 | 0.810 |
| NYHA class ≥III | 1.85 | 1.08, 1.47 | **0.004** |
| Atrial fibrillation | 1.40 | 1.03, 1.34 | **0.016** |
| LAVi ≥60 mL/m^2^ | 0.99 | 0.87, 1.13 | 0.950 |
| PASP ≥50 mmHg | 1.17 | 0.96, 1.23 | 0.210 |
| LVESD ≥40 mm | 0.92 | 0.68, 1.24 | 0.560 |
| LVEF <60% | 0.90 | 0.69, 1.18 | 0.430 |
|  | | | |
| **Bold** values indicate statistical significance. *LAVi = left atrial volume index; LVEF = left ventricular ejection fraction; LVESD = left ventricular end-systolic diameter; MIDA = Mitral Regurgitation International Database; NYHA = New York Heart Association; PASP = pulmonary artery systolic pressure* | | | |

## Supplemental Table 12. Cox Proportional Hazards Model for the Primary Endpoint Using Isolated Modified MIDA Score Parameters and NT-proBNP

| **Variable** | **Multivariable Analysis** | | |
| --- | --- | --- | --- |
|  | **Hazard Ratio** | **95% Confidence Interval** | **P value** |
| Age ≥65 years | 0.99 | 0.57, 1.71 | 0.970 |
| Baseline NYHA ≥III | 1.67 | 1.10, 2.53 | **0.015** |
| Atrial fibrillation | 1.31 | 1.00, 1.73 | 0.053 |
| LAVi ≥60 mL/m^2^ | 0.96 | 0.74, 1.25 | 0.780 |
| PASP ≥50 mmHg | 1.08 | 0.84, 1.39 | 0.550 |
| LVESD ≥40 mm | 0.91 | 0.67, 1.23 | 0.530 |
| LVEF <60% | 0.84 | 0.64, 1.10 | 0.210 |
| NT-proBNP (log) | 1.20 | 1.07, 1.34 | **0.002** |
|  | | | |
| **Bold** values indicate statistical significance. *LAVi = left atrial volume index; LVEF = left ventricular ejection fraction; LVESD = left ventricular end-systolic diameter; MIDA = Mitral Regurgitation International Database; NYHA = New York Heart Association; NT-proBNP = N-terminal pro-B-type natriuretic peptide; PASP = pulmonary artery systolic pressure* | | | |

## Supplemental Table 13. Cox Proportional Hazards Model for the Primary Endpoint Using Isolated Original MIDA Score Parameters

| **Variable** | **Multivariable Analysis** | | |
| --- | --- | --- | --- |
|  | **Hazard Ratio** | **95% Confidence Interval** | **P value** |
| Age ≥65 years | 1.17 | 0.68, 2.01 | 0.570 |
| Baseline NYHA ≥II | 0.81 | 0.29, 2.25 | 0.680 |
| Atrial fibrillation | 1.44 | 1.10, 1.87 | **0.008** |
| LA diameter | NA | NA | NA |
| PASP ≥50 mmHg | 1.23 | 0.96, 1.57 | 0.110 |
| LVESD ≥40 mm | 0.94 | 0.70, 1.26 | 0.660 |
| LVEF <60% | 0.90 | 0.69, 1.18 | 0.460 |
|  | | | |
| **Bold** values indicate statistical significance. *LAVi = left atrial volume index; LVEF = left ventricular ejection fraction; LVESD = left ventricular end-systolic diameter; MIDA = Mitral Regurgitation International Database; NYHA = New York Heart Association; PASP = pulmonary artery systolic pressure* | | | |

## Table 14. Cox Proportional Hazards Model for the Primary Endpoint using Isolated Original MIDA Score Parameters and NT-proBNP

| **Variable** | **Multivariable Analysis** | | |
| --- | --- | --- | --- |
|  | **Hazard Ratio** | **95% Confidence Interval** | **P value** |
| Age ≥65 years | 1.05 | 0.60, 1.81 | 0.870 |
| Baseline NYHA ≥II | 0.69 | 0.24, 1.93 | 0.470 |
| Atrial fibrillation | 1.32 | 1.01, 1.73 | **0.044** |
| LA diameter | NA | NA | NA |
| PASP ≥50 mmHg | 1.10 | 0.85, 1.42 | 0.450 |
| LVESD ≥40 mm | 0.93 | 0.69, 1.25 | 0.610 |
| LVEF <60% | 0.83 | 0.63, 1.09 | 0.180 |
| NT-proBNP (log) | 1.23 | 1.10, 1.38 | **<0.001** |
|  | | | |
| **Bold** values indicate statistical significance. *LA = left atrium; LVEF = left ventricular ejection fraction; LVESD = left ventricular end-systolic diameter; MIDA = Mitral Regurgitation International Database; NYHA = New York Heart Association; NT-proBNP = N-terminal pro-B-type natriuretic peptide; PASP = pulmonary artery systolic pressure* | | | |
